# How does rest intolerance affect physical exercise? A dual-pathway mediation model of exercise adherence and psychological resilience

**DOI:** 10.3389/fpsyg.2025.1723996

**Published:** 2026-01-06

**Authors:** Ding-you Zhang, Hu Lou, Jun Liu, Bo Li

**Affiliations:** Institute of Sports Science, Nantong University, Nantong, China

**Keywords:** dual-path chain mediation, exercise motivation, physical exercise, rest intolerance, university students

## Abstract

**Objective:**

This study investigated the influence of rest intolerance on physical exercise levels among university students. It examined the independent mediating roles of exercise adherence and psychological resilience, as well as their serial mediation through exercise motivation.

**Methods:**

A survey was administered to 24,979 university students from 155 institutions across 17 provinces in China. The survey included the Rest Intolerance Scale-8 (RIS-8), Exercise Adherence Scale, Adolescent Psychological Resilience Scale, Exercise Motivation Profile for Physical Activity (MPAM-R), and Physical Activity Rating Scale (PARS-3). Data were analysed using SPSS 27.0 with the PROCESS macro, employing 5,000 bias-corrected bootstrap resamples to test mediation effects.

**Results:**

Correlation analysis revealed that rest intolerance exhibited significant positive correlations with exercise adherence (*r* = 0.059) and exercise motivation (*r* = 0.006), whilst showing significant negative correlations with physical exercise (*r* = −0.016) and psychological resilience (*r* = −0.196). Exercise adherence, psychological resilience, and exercise motivation all demonstrated significant positive correlations with physical exercise. Logistic regression analysis indicated that rest intolerance significantly reduced the likelihood of achieving higher exercise levels (moderate vs. low: OR = 0.992; high vs. low: OR = 0.987). Exercise adherence emerged as the strongest predictor (OR = 1.126–1.247), whilst psychological resilience exhibited inconsistent minor association across exercise level comparisons. Gender, year group, and BMI also significantly influenced the attainment of exercise levels. Mediation analysis revealed that rest intolerance had no significant total association on physical exercise (Effect = −0.021), yet exhibited a significant direct negative association (*β* = −0.063, *p* < 0.001), demonstrating a classic suppression effect. Exercise adherence produced a significant positive mediation effect (Effect = 0.066), while psychological resilience alone showed no significant mediation. Both chain mediations were significant but directionally opposite: ‘rest intolerance → exercise adherence → exercise motivation → physical exercise’ exhibited negative chain mediation (Effect = −0.020); ‘rest intolerance → psychological resilience → exercise motivation → physical exercise’ exhibited positive chain mediation (Effect = 0.003). The total indirect effect was significant (Effect = 0.042).

**Conclusion:**

Rest intolerance exerts a direct inhibitory association on physical exercise, yet its indirect pathways exhibit dual-directional influences through ‘behavioural persistence promotion’ and ‘motivation depletion inhibition’, thereby obscuring the overall association. Exercise adherence serves as the most critical positive mediator, while diminished motivation quality constitutes a negative chain mechanism. Interventions targeting university students with high rest intolerance must concurrently guide healthy persistence strategies and enhance motivational autonomy alongside psychological resources.

## 1 Introduction

The “Medium- and Long-Term Youth Development Plan (2016–2025),” issued by the Central Committee of the Communist Party of China and the State Council, mandates that at least 90% of young people meet physical fitness standards and explicitly requires ensuring adequate physical education class hours and extracurricular exercise time (The Central Committee of the Communist Party of China and the State Council, 2017). However, despite this national emphasis, insufficient physical activity remains a prevalent issue among university students, exacerbated by academic pressures and employment concerns in the context of rapid socio-economic development (Guthold et al., 2018; Tan, 2020). A World Health Organization (WHO) report indicates that over 80% of school-aged adolescents worldwide do not meet the recommended 1 h of daily physical activity, with rates of 85% among girls and 78% among boys (WHO, 2019). This lack of physical activity is associated with declining physical fitness, an increased risk of chronic diseases, impaired emotional regulation, challenges in social adaptation, and a reduced long-term developmental potential (Goje et al., 2014). However, traditional research has predominantly addressed insufficient exercise from perspectives such as stress, environment, and motivation. Little exploration has been conducted into whether the phenomenon of “rest intolerance”—which has gained prominence in recent years—contributes to inadequate physical exercise among university students. Therefore, identifying key factors that influence physical activity behaviour in university students and developing targeted interventions is both theoretically valuable and practically urgent.

Rest intolerance, as a multidimensional concept, refers to the negative psychological experience whereby individuals, during non-productive rest, experience shame, guilt, self-criticism, or anxiety due to concerns that their rest may be perceived as laziness, a lack of self-discipline, or a waste of time (Wang et al., 2025). Its psychological structure comprises four interrelated core dimensions: negative emotions, social comparison, obsessive thoughts, and cognitive biases (Wang et al., 2025). Negative emotions constitute the most direct psychological experience, manifesting as shame, guilt, and self-deprecation during rest. Social comparison exacerbates unease by focusing on peers’ productive activities (Wang et al., 2025). Regarding influencing factors, anxiety and depressive symptoms among Chinese university students, alongside high perceived stress, amplify shame. Online social media addiction exhibits a significant positive correlation with rest intolerance (*β* = 0.32). Conversely, higher health literacy (*β* = −0.17) and greater self-compassion are correlated with reduced shame. Field-dependent personalities, being more concerned with external evaluation, tend to experience stronger shame (Avcı, 2025). At the cultural level, East Asia’s diligence-centric value orientation is characterised by a pervasive emphasis on productivity ethics, which stresses the connection between effort and moral worth. Its cultural specificity manifests in viewing diligence not merely as personal virtue but as fulfilment of familial, collective, and societal responsibilities, thereby rendering rest more susceptible to moral labelling as slackness, selfishness, or dereliction of duty. Concepts such as “heaven rewards diligence” and “hard work compensates for shortcomings” have long permeated Chinese education and socialisation processes, conditioning individuals to tightly bind their sense of self-worth to a state of perpetual labour or study (Liu and Du, 2025). The self-discipline, technological discipline, and institutional discipline faced by university students collectively create an environment that intensifies guilt associated with rest. During high-pressure academic periods, such as exam weeks or thesis deadlines, or when observing peers engaging in productive activities while others rest, feelings of shame are directly triggered and amplified, implicitly stigmatising rest as immoral (Zhao et al., 2025). The theoretical underpinnings of rest intolerance are rooted in self-determination theory and conservation of resources theory. From a self-determination perspective, rest intolerance fundamentally represents the suppression of autonomy needs—individuals perceive rest as behaviour at odds with societal expectations and contrary to their intrinsic desires (Ryan and Deci, 2000). Resource conservation theory posits that individuals with high rest intolerance tend to compensate for rest time through excessive dedication to study or work, viewing sustained action as a resource that safeguards their sense of social worth. These phenomena not only impact individual psychological well-being but may also exacerbate existing psychological pathologies by intensifying stress and anxiety. Its characteristics manifest primarily through cognitive, emotional, and behavioural dimensions (Hobfoll et al., 2016).

These characteristics distinguish rest intolerance from other related constructs: it differs from general shame, which pertains to overall self-evaluation and arises from diverse situations, whereas the shame associated with rest intolerance is particular to rest and is triggered solely when action is suspended (Park, 2023); Unlike perfectionism, where shame stems from failing to meet high standards or making errors, rest intolerance does not depend on performance benchmarks; even when objectives are achieved, one may still feel undeserving of rest itself (刘, 2025); It also differs from workaholism, where the driving force is a compulsive desire to work or an inability to disengage from tasks. Those with rest intolerance may not necessarily wish to work, but simply cannot tolerate rest (Clark et al., 2016); It further differs from performance anxiety, which stems from fear of failure or evaluation. Rest intolerance focuses not on the consequences of performance, but on the negative moralization of the act of resting itself (Hall et al., 1998). Thus, rest intolerance constitutes a distinct construct whose mechanisms stem not solely from anxiety, overcommitment, or compulsive motivation, but are closely intertwined with cultural values, internalised norms, and self-imposed moral demands. Given its pervasive influence on behavioural choices across academic, occupational, and sporting contexts, incorporating rest intolerance as an independent psychological variable into physical exercise research will enhance our understanding of university students’ decision-making patterns and the logic behind their health behaviours regarding exercise versus recovery.

Physical exercise, as an activity possessing both restorative attributes and health benefits, exhibits a distinctive association with rest intolerance (Sun et al., 2023). Although studies directly examining the relationship between rest intolerance and physical exercise behaviour remain scarce, existing empirical evidence provides essential support for this research hypothesis at both the health and emotional mechanism levels. For instance, rest intolerance exhibits significant correlations with adverse indicators such as anxiety, stress, emotional distress, and sleep disturbances (Avcı, 2025). These psychological and physiological burdens have been demonstrated across extensive sports psychology research to diminish individuals’ motivation and engagement in physical activity. Concurrently, network analysis among Chinese nursing students identified rest intolerance as a pivotal node linking emotional burden to restricted health behaviours, suggesting it may diminish physical participation by intensifying negative emotional experiences (Zhao et al., 2025). Research on the Chinese University Students’ Rest Intolerance Scale further indicates that most nodes in the questionnaire’s axial coding pertain to negative emotions, signifying their centrality in conceptualising rest intolerance (Wang et al., 2025). Extensive research has confirmed that regular physical exercise not only significantly enhances physical health but also exerts significant psychological protective association by reducing negative mental states such as anxiety, depression, stress, and emotional exhaustion. Exercise is regarded as a prototypical positive behavioural pattern that promotes emotional regulation, enhances self-efficacy, and facilitates physical and mental recovery, with these benefits being particularly pronounced in youth and university student samples (Zhou et al., 2022; Chan Kim and Abbas, 2024; Naqvi and Prasad, 2025; Cai, 2022; Guiping et al., 2013). Therefore, given that rest intolerance frequently co-occurs with heightened emotional distress, perceived stress, and self-critical tendencies, physical exercise holds potential as a vital resource to counteract or mitigate the negative consequences of rest intolerance. Accordingly, the following hypothesis is proposed: H1. Rest intolerance significantly and negatively predicts physical exercise engagement among university students.

As a key variable in the domain of physical exercise engagement, exercise adherence denotes an individual’s willingness and capacity to maintain regular physical activity despite encountering difficulties, temptations, or obstacles. It serves as a crucial indicator for sustaining healthy behaviours (Dishman, 1988). Self-Determination Theory (SDT) posits that behavioural persistence hinges upon the fulfilment of three fundamental psychological needs: autonomy, competence, and relatedness (Deci and Ryan, 2000; Matsumoto and Takenaka, 2022). However, rest intolerance may undermine an individual’s sense of autonomy, leading them to perceive exercise as an “atonement” or “compensatory” act, thereby lacking intrinsic motivational support (Van Roie et al., 2015; Rodrigues et al., 2018). In such circumstances, individuals with high rest intolerance may adopt a coerced persistence pattern, where exercise serves to alleviate guilt arising from rest. This manifests as superficial exercise adherence lacking a stable motivational foundation. Whilst such compensatory persistence may sustain physical activity in the short term, it struggles to promote sustained long-term physical exercise levels. Based on the aforementioned mechanisms, exercise adherence may mediate the relationship between rest intolerance and physical exercise. Hence, hypothesis H2 is proposed: Exercise adherence mediates the relationship between rest intolerance and physical exercise levels among university students.

Secondly, psychological resilience—as an individual’s capacity for positive adaptation and rapid recovery in the face of adversity—constitutes a vital psychological resource for sustaining healthy behaviours (Sisto et al., 2019; Baona et al., 2012; Shuo et al., 2022). According to the Conservation of Resources Theory (COR), individuals strive to protect and accumulate resources to cope with stressful situations (Hobfoll, 1989; Davydov et al., 2010). Rest intolerance itself constitutes a chronic psychological stressor, leading to adverse experiences such as self-deprecation, anxiety, and depression, thereby depleting psychological resources and diminishing psychological resilience (Tugade and Fredrickson, 2004). When psychological resilience is compromised, individuals find it more challenging to maintain regular exercise and derive positive experiences from physical activity. Conversely, highly resilient individuals can mitigate the effects of rest intolerance through positive emotion regulation and seeking support, thereby maintaining relatively stable exercise behaviour (Tugade and Fredrickson, 2004). Thus, psychological resilience may mediate the relationship between rest intolerance and physical exercise, leading to the following hypothesis: H3: Psychological resilience mediates the relationship between rest intolerance and physical exercise among university students.

**Figure 1:**
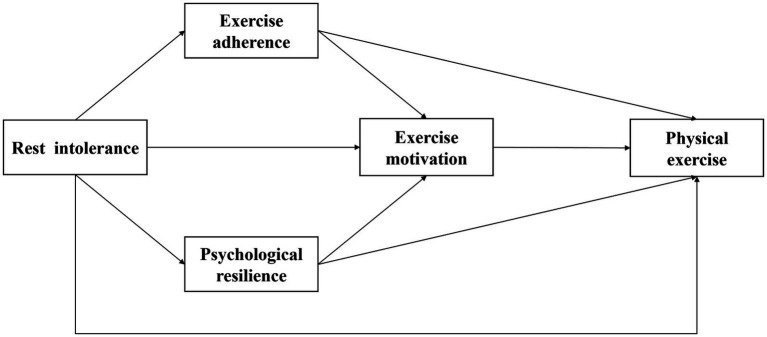
Schematic diagram of the mediator model.

Furthermore, exercise motivation refers to the intrinsic driving force behind an individual’s participation in exercise behaviour, directly influencing their choice, exercise adherence, and level of effort in such activities (Markland and Ingledew, 1997). Within the framework of self-determination theory, exercise motivation is a key factor determining whether an individual can sustain long-term exercise habits (Teixeira et al., 2012; Wilson et al., 2008). Exercise adherence can foster intrinsic motivation by enhancing competence and self-efficacy, while psychological resilience provides the psychological foundation for internalising motivation through emotional regulation and resource accumulation (Dishman et al., 2021). However, compensatory persistence induced by rest intolerance often lacks autonomy, failing to generate enjoyment or satisfaction. Instead, it may increase fatigue, diminish self-efficacy, and weaken the foundation of intrinsic motivation. Similarly, psychological resource depletion stemming from rest intolerance further diminishes the motivational quality (Rhodes et al., 2021). Thus, rest intolerance may influence physical exercise levels through two chained pathways—‘exercise adherence → motivation’ and ‘psychological resilience → motivation’—forming a multi-layered transmission mechanism from stressor to behavioural outcome. Based on this, the present study proposes the following chained mediation hypotheses: H4: Exercise adherence and exercise motivation mediate the relationship between rest intolerance and university students’ physical exercise levels; H5: Psychological resilience and exercise motivation mediate the relationship between rest intolerance and university students’ physical exercise levels (Figure 1).

## 2 Method

### 2.1 Research subjects

The participants in this survey were students enrolled in standard higher education institutions within mainland China, with institutions identified according to the Ministry of Education’s “National List of Standard Higher Education Institutions (as of June 20, 2024).” This study employed epidemiological survey methods. The investigation included 17 provinces: Jiangsu, Shanghai, Shandong, Jilin, Henan, Sichuan, Chongqing, Guizhou, Yunnan, Shaanxi, Gansu, Qinghai, Ningxia, Xinjiang, Guangxi, Inner Mongolia, and Hainan. Following the principles of stratified cluster sampling, representative samples were drawn from these provinces. The final sample encompassed 155 higher education institutions: 42 in Category I (provincial capital cities), 73 in Category II (municipalities with average socio-economic status), and 40 in Category III (municipalities with relatively lower socio-economic conditions).

Data collection was conducted online, with researchers and supervisors present during the data entry process. Participants received informed consent forms at the study’s outset, which clearly outlined research objectives, methodologies, potential risks, and participant rights, ensuring voluntary and fully informed participation. All participants were told that the questionnaire would take approximately 12 min to complete and that they could withdraw at any time without repercussions. A pre-survey was conducted to refine the questionnaire design based on the feedback received. Responses were collected anonymously, and sample data remained confidential to minimise self-reporting bias. This study received approval from the ethics committee, and all participants provided informed consent.

To ensure data quality, this study rigorously verified the authenticity and validity of the recovered online questionnaires in accordance with the data cleansing principles of the primary database. A total of 36,756 questionnaires were collected, and after a four-step screening process, 24,979 valid questionnaires were ultimately retained. The specific procedure is as follows:

Step 1: Exclude questionnaires where the full name of the institution was unidentifiable (1,324 removed).

Specific questionnaires contained garbled text, abbreviations, codes, invalid characters, or illogical entries in the ‘School Name’ field. Such responses typically indicated insufficient attention or invalid content, warranting exclusion. Remaining after screening: 35,432 questionnaires.

Step 2: Exclusion of questionnaires where age fell outside the target demographic range (15–30 years) (618 excluded).

Given this study’s focus on university students, records indicating ages below 15 or above 30 were excluded to prevent non-target demographics from skewing data distribution. Remaining after exclusion: 34,814.

Step 3: Removal of questionnaires with ≥21 consecutive identical responses (2,817 excluded).

Such mechanically consistent responses (e.g., selecting the same option for all questions) were deemed indicative of low-quality responses, potentially reflecting non-reading or automated completion. Following the central database methodology, consecutive identical selections were used as a core quality metric for exclusion. Remaining after exclusion: 31,997 responses.

Step 4: Exclusion of anomalous samples based on completion duration (7,018 responses excluded).

The distribution of completion times for the remaining questionnaires was statistically analysed. After calculating the average completion time, samples falling within the shortest 5% (excessively brief) and longest 5% (excessively lengthy) of the distribution were excluded. Excessively brief completion times suggested a failure to read the questions, while excessively lengthy times indicated potential interruptions or discontinuities in the completion process. After exclusion, the remaining sample size was 24,979.

The detailed information of the final sample is shown in table (Table 1).

**Table 1:** Sample distribution table.

Variable	Classification	*n*	%
Gender	Male	9,796	39.2
	Female	15,183	60.8
Grade	First Grade	14,599	58.4
	Second Grade	8,108	32.5
	Third Grade	1,640	6.6
	Fourth Grade	632	2.5
Age	18–20 years old	17,429	69.8
	20–22 years old	5,044	20.2
	22–24 years old	635	2.5
	Over 24 years old	147	0.6
BMI	<18.5	4,480	17.9
	18.5–25	16,096	64.4
	25–30	2,594	10.4
	>30	1,809	7.2
Total		24,979	100

### 2.2 Research tools

#### 2.2.1 Rest intolerance

In this study, university students’ rest intolerance was measured using the Rest Intolerance Scale-8 (RIS-8) developed by Wang et al. (2025). This scale comprises eight items, ranging from “When I rest or engage in leisure activities, I feel guilty” to “I believe time off should be spent on more worthwhile pursuits.” Scoring employs a five-point Likert scale, with one representing “strongly disagree” and five representing “strongly agree.” Higher scores indicate greater rest intolerance. This scale was developed for Chinese university students and has been validated within this population. The scale demonstrated a Cronbach’s *α* of 0.89, with CFI = 0.99 (>0.9), GFI = 0.98 (>0.8), and RMSEA = 0.063 (<0.08). The Cronbach’s *α* in this study was 0.93, indicating good reliability and validity and confirming the scale as a sound research instrument for measuring rest intolerance (Wang et al., 2025).

#### 2.2.2 Physical exercise

The level of physical exercise among university students in this study was measured using the Physical Activity Rating Scale-3 (PARS-3), developed by Japanese scholar Hashimoto Kimio in 1990 and translated and revised by Chinese scholar Liang (1994). The PARS-3 assesses physical exercise volume across three dimensions: intensity, frequency, and duration per session. Each dimension comprises five options measuring participation levels, scored from 1 to 5. The total score ranges from 0 to 100, with higher scores indicating greater physical activity levels. Following revision, this scale has been extensively applied among university students in China (Wu et al., 2022; Chen and Yu, 2015; Zhu et al., 2014). In the original Chinese-language study, the PARS-3 yielded a Cronbach’s *α* coefficient of 0.78, with GFI = 0.93, AGFI = 0.92, CFI = 0.88, and RMSEA = 0.06, as well as a retest reliability of 0.82. In the present study, the scale demonstrated a Cronbach’s *α* coefficient of 0.79, indicating that it is a reliable and valid research instrument.

#### 2.2.3 Exercise adherence

In this study, exercise adherence among university students was measured using the Exercise Adherence Scale, developed in 2016 by Chinese scholar Gu Chunqiang for Chinese exercise participants (Gu, 2016). This scale comprises three dimensions—exercise behaviour, effort commitment, and emotional experience—totalling 15 items. A five-point Likert scale was employed, ranging from 1 (“completely disagree”) to 5 (“completely agree”). Higher scores indicate greater exercise adherence. In the original scale study, the total scale reliability was 0.9547, with fit indices of *χ*^2^/df = 2.896, CFI = 0.945, GFI = 0.901, and RSEA = 0.069 (Gu, 2016). In the present study, the Cronbach’s *α* coefficient for this scale was 0.94, demonstrating its suitability as a general research instrument for assessing exercise participation (Figure 1 and Table 1).

#### 2.2.4 Psychological resilience

In this study, psychological resilience among university students was measured using the Adolescent Psychological Resilience Scale developed by Hu Yueqin. This instrument was selected because university students remain within the adolescent developmental stage, and psychological resilience, as a relatively stable psychological trait, exhibits structural continuity and measurement consistency during the transition from adolescence to early adulthood. Furthermore, this scale has undergone extensive validation within Chinese university populations (Liu, 2020; Hu, 2019), making it an appropriate research tool for assessing psychological resilience among university students (Hu, 2008). The scale comprises 27 items across five dimensions: goal focus, emotional regulation, positive cognition, family support, and interpersonal assistance. It employs a 5-point Likert scale, ranging from 1 = ‘completely disagree’ to 5 = ‘completely agree’, yielding total scores between 27 and 135 points (Hu, 2008). The total score is calculated across all items, with higher scores indicating greater psychological resilience. In the original study, the total scale had an *α* of 0.85, CFI of 0.92, and RMSEA of 0.07, demonstrating a good confirmatory factor analysis fit. In this study, the Cronbach’s *α* coefficient for this scale was 0.78. It serves as a sound instrument for assessing psychological resilience among university students.

#### 2.2.5 Exercise motivation

The Motives for Physical Activity Measure (MPAM-R), developed by Ryan et al., is a commonly used instrument for studying exercise motivation. The Motives for Physical Activity Measure–Revised (MPAM-R) was developed by Ryan et al. (1997), targeting adult exercisers (Liu, 2020). Grounded in cognitive evaluation theory and self-determination theory, this scale comprises five dimensions of exercise motivation. External motivations include health promotion and appearance enhancement, while internal motivations encompass enjoyment, competence development, and social interaction. A five-point Likert scale is used, ranging from “strongly disagree” to “strongly agree,” with higher scores indicating greater exercise motivation. The Chinese version was translated and revised by Chinese scholars, including Chen Shanping, and underwent simplification in 2006. Its applicability has been validated among university students in China (Chen et al., 2006). To streamline measurement, Chen et al. condensed the MPAM-R Chinese version into a 15-item simplified scale. The simplified version of the motivation scale employed in this study yielded the following fit indices: GFI = 0.93, AGFI = 0.92, CFI = 0.88, RMSEA = 0.06, and Cronbach’s *α* = 0.92. Within this study, the Cronbach’s *α* coefficient for the simplified scale was 0.90, indicating a high level of internal consistency.

### 2.3 Data processing and statistical methods

Data processing primarily utilised SPSS 27.0 and Excel software, divided into key stages: 【1】 Preliminary data handling via Excel for responses collected through the Questionnaire Star platform, including re-measurement or removal of incomplete or anomalous data. 【2】 Application of SPSS for common method bias detection to ensure research accuracy. 【3】 Descriptive data analysis was conducted on the collected student data. Pearson’s correlation analysis was employed to examine the relationships between university students’ rest intolerance, exercise adherence, psychological resilience, exercise motivation, and physical exercise levels. Collinearity checks were performed on these variables to prevent issues with multicollinearity. 【4】 Considering that the outcome variable—physical exercise level (PARS-3 score)—is continuous yet may not fully satisfy the residual normality assumption for linear regression, physical exercise was recoded into a categorical variable with clinical or practical significance. This facilitates interpretation of results from the perspective of ‘achieving sufficient exercise levels,’ rendering conclusions more intuitive. Specifically, we converted physical exercise levels into a three-level ordinal variable based on the commonly used classification standard of the Physical Activity Level Scale: 1 = ‘low exercise’ (≤19 points), 2 = ‘moderate exercise’ (20–42 points), 3 = ‘high exercise’ (≥43 points) (Liang, 1994). Subsequently, the independent variable ‘rest intolerance’ and mediating variables were incorporated to construct an ordinal logistic regression model. This model estimated parameters through two cumulative comparisons. Results were reported as unstandardised regression coefficients (B), odds ratios (OR), and their 95% confidence intervals (CI). An OR value below 1 indicates the variable acts as an impediment to achieving higher physical activity levels, while a value above 1 signifies a facilitating factor. 【5】 To examine the chained mediation model incorporating multiple mediators, regression analysis and mediation association testing were conducted using the SPSS PROCESS macro. Based on research hypotheses (H2 to H5), the model included two parallel mediation pathways (rest intolerance → exercise adherence → physical exercise; rest intolerance → psychological resilience → physical exercise) and two chained mediation pathways (rest intolerance → exercise persistence → exercise motivation → physical exercise; rest intolerance → psychological resilience → exercise motivation → physical exercise). To simultaneously test these pathways, the PROCESS macro’s Model 80 was employed. According to Hayes’ model definition, Model 80 enables more precise estimation of competing or offsetting mechanisms within complex psychological processes by controlling for cross-path influences, compared to splitting multiple mediations into separate models (Hayes, 2017). Consequently, this model structure perfectly aligns with the proposed ‘parallel + serial’ dual-path hypothesis. It constitutes the essential statistical method for examining the key mechanisms through which rest intolerance influences physical exercise. Association testing was conducted concurrently using bias-corrected Bootstrap sampling with 5,000 repeated samples. Results are reported as unstandardized regression coefficients (*β*), standard errors (SE), 95% bias-corrected confidence intervals (95% CI), and coefficients of determination (*R*^2^). Path coefficients were deemed significant if their 95% CI did not encompass zero.

## 3 Results

### 3.1 Common method bias

In this study, data collection relied on subjective scales completed by the participants. To mitigate common method bias, exploratory factor analysis (EFA) was conducted on all questionnaire items related to rest intolerance, exercise adherence, psychological resilience, exercise motivation, and physical exercise variables using Harman’s single-factor test after data collection. Results indicated extraction of nine principal components with eigenvalues exceeding 1, with the largest factor explaining 27.387% of the variance—below the conventional 40% threshold (Podsakoff et al., 2003). Therefore, common method bias was not a significant concern in this study.

### 3.2 Descriptive analysis and correlation analysis

Table 2 reports the mean, standard deviation, VIF, and Pearson correlation coefficients for each variable. Results indicate that rest intolerance and exercise adherence (*r* = 0.059, *p* < 0.001) and exercise motivation (*r* = 0.006, *p* < 0.001) exhibit significant positive correlations, whilst showing significant negative correlations with psychological resilience (*r* = −0.196, *p* < 0.001) and physical exercise (*r* = −0.016, *p* < 0.001, *p* < 0.01) and physical exercise (*r* = −0.016, *p* < 0.01). Exercise adherence showed significant positive correlations with psychological resilience (*r* = 0.451, *p* < 0.001), exercise motivation (*r* = 0.436, *p* < 0.001), and physical exercise (*r* = 0.426, *p* < 0.001). Psychological resilience and exercise motivation (*r* = 0.220, *p* < 0.001) and physical exercise (*r* = 0.231, *p* < 0.001) both showed significant positive correlations. Exercise motivation and physical exercise also exhibited a significant positive correlation (*r* = 0.207, *p* < 0.001). Concurrently, collinearity analysis revealed that all variables exhibited a coefficient of less than 4, indicating no collinearity issues in this study.

**Table 2:** Descriptive statistics, VIF and Pearson correlation table.

Variables	*M*	SD	Tolerance	VIF	1	2	3	4
1. Rest intolerance	32.546	9.978	0.933	1.072	/			
2. Exercise adherence	50.503	9.230	0.562	1.780	0.059**	/		
3. Psychological resilience	95.565	13.598	0.746	1.340	−0.196**	0.451**	/	
4. Exercise motivation	18.575	3.380	0.809	1.237	0.064**	0.436**	0.220**	/
5. Physical exercise	17.981	19.856	0.784	1.275	−0.016*	0.462**	0.231**	0.207**

*p < 0.01, **p < 0.001 (double tailed).

### 3.3 Regression analysis

Table 3 reports the ordinal logistic regression model with physical exercise intensity level (low/moderate/high) as the dependent variable. Results indicate that rest intolerance significantly negatively predicts exercise volume increase (medium vs. low: *B* = −0.008, OR = 0.992, 95% CI = [0.988, 0.996]; high vs. low: *B* = −0.013, OR = 0.987, 95% CI = [0.983, 0.991]). Psychological resilience positively predicted exercise volume in the comparison between moderate and low exercise levels (*B* = 0.023, OR = 1.024, 95% CI = [1.021, 1.026]), and also showed positive predictive value in the comparison of high versus low exercise intensity (*B* = 0.029, OR = 1.030, 95% CI = [1.027, 1.033]). Exercise adherence emerged as the strongest predictor of progression in exercise intensity levels. Each one-point increase in adherence was associated with a 12.6% greater likelihood of reaching moderate intensity (*B* = 0.119, OR = 1.126, 95% CI = [1.116, 1.137]) and a 24.7% greater likelihood of reaching vigorous intensity (*B* = 0.220, OR = 1.247, 95% CI = [1.231, 1.262]). The gender association was significant: males exhibited only 35.8% of females’ advantage at moderate exercise intensity (*B* = −1.027, OR = 0.358, 95% CI = [0.333, 0.385]), and merely 14.8% at vigorous intensity (*B* = −1.908, OR = 0.148, 95% CI [0.134, 0.164]). Grade level showed a positive predictive association only in the comparison between high and low exercise levels (*B* = 0.221, OR = 1.247, 95% CI = [1.178, 1.321]), with no significant difference observed between moderate and low exercise levels (*B* = −0.030, OR = 0.971, 95% CI = [0.923, 1.020]). BMI exhibited a marginally negative predictive association (moderate vs. low: *B* = −0.008, OR = 0.992, 95% CI = [0.986, 0.998]; high vs. low: *B* = −0.018, OR = 0.982, 95% CI = [0.974, 0.990]).

**Table 3:** Logistic regression analysis of predictive factors for different levels of physical exercise.

Compare levels	Predictor variable	*B*	SE	Wald *ꭓ*^2^	*p*	OR	95CI%	
							Lower	Upper
Medium exercise vs. Low exercise	Rest intolerance	−0.008	0.002	18.461	<0.001	0.992	0.988	0.996
	Psychological resilience	0.023	0.002	344.463	<0.001	1.024	1.021	1.026
	Exercise motivation	−0.150	0.012	146.035	<0.001	0.861	0.840	0.882
	Exercise adherence	0.119	0.005	608.358	<0.001	1.126	1.116	1.137
	Gender	−1.027	0.037	750.381	<0.001	0.358	0.333	0.385
	Year group	−0.030	0.026	1.362	0.243	0.971	0.923	1.020
	BMI	−0.008	0.003	6.402	0.011	0.992	0.986	0.998
High exercise vs. Low exercise	Rest intolerance	−0.013	0.002	35.663	<0.001	0.987	0.983	0.991
	Psychological resilience	0.029	0.001	423.303	<0.001	1.030	1.027	1.033
	Exercise motivation	−0.242	0.016	223.953	<0.001	0.785	0.760	0.810
	Exercise adherence	0.220	0.006	1,211.922	<0.001	1.247	1.231	1.262
	Gender	−1.908	0.051	1,381.623	<0.001	0.148	0.134	0.164
	Year group	0.221	0.029	56.621	<0.001	1.247	1.178	1.321
	BMI	−0.018	0.004	20.444	<0.001	0.982	0.974	0.990

### 3.4 Mediation effect test

To explore the complex interaction mechanisms among variables, a bias-corrected Bootstrap method was employed to test the multiple mediation model. The results in Table 4 indicate that the total effect was non-significant (Effect = −0.021, 95% CI [−0.044, 0.002]), while the direct effect was significant (Effect = −0.063, 95% CI [−0.085, −0.042]), while the total indirect effect was significant (Effect = 0.042, 95% CI [0.028, 0.056]). This indicates that the mediating pathway obscures the negative impact of rest intolerance on physical exercise, revealing a pronounced masking effect within the model. Indirect effect analyses showed that the pathway ‘rest intolerance → exercise adherence → physical exercise’ exhibited a significant positive mediation (Effect = 0.066, 95% CI [0.048, 0.084]), while the pathway ‘rest intolerance → psychological resilience → physical exercise’ showed a non-significant positive mediation (Effect = 0.002, 95% CI [−0.004, 0.007]), while ‘rest intolerance → exercise adherence → exercise motivation → physical exercise’ (Effect = −0.020, 95% CI [−0.026, −0.014]) exhibited a significant negative chain mediation. The chain mediation from ‘rest intolerance → psychological resilience → exercise motivation → physical exercise’ (Effect = 0.003, 95% CI [0.002, 0.003]) demonstrated a significant positive chain mediation.

**Table 4:** Bootstrap test results for mediating effects.

Effect type	Paths	Effect	BootSE	LLCI	ULCI
Total effect	/	−0.021	0.012	−0.044	0.002
Direct effect	Rest intolerance → physical exercise	−0.063	0.011	−0.085	−0.042
Total indirect effect	/	0.042	0.007	0.028	0.056
Indirect effect 1	Rest intolerance → exercise adherence → physical exercise	0.066	0.009	0.048	0.084
Indirect effect 2	Rest intolerance → psychological resilience → physical exercise	0.002	0.003	−0.004	0.007
Indirect effect 3	Rest intolerance → exercise motivation → physical exercise	−0.008	0.001	−0.010	−0.005
Indirect effect 4	Rest intolerance → exercise adherence → exercise motivation → physical exercise	−0.020	0.003	−0.026	−0.014
Indirect effect 5	Rest intolerance → psychological resilience → exercise motivation → physical exercise	0.003	0.000	0.002	0.003

## 4 Discussion

### 4.1 The direct impact of rest intolerance on physical exercise levels

This study, through large-sample surveys and regression analysis, found that rest intolerance exerts a significant negative predictive association on university students’ physical exercise levels (*β* = −0.063, *p* < 0.001), thereby validating Hypothesis H1. This finding aligns with Wang’s research, which demonstrated that individuals with high rest intolerance are more prone to avoid rest due to guilt, consequently reducing time allocated to physical exercise (Wang et al., 2025). This study found no evidence that rest intolerance promotes physical exercise through an “overcompensation” mechanism. This may stem from the interaction between cultural context and sample characteristics: within East Asian cultural frameworks, university students commonly face pressures of grade competition and involvement. To achieve higher grades and more outstanding academic performance, university students may devote greater energy and time to work and study as a means of alleviating stress and anxiety, rather than engaging in physical exercise to promote healthy behaviours (Gu et al., 2021). This study found that the overall association of rest intolerance on physical exercise was not significant (Effect = −0.021, 95% CI [−0.044, 0.002]). However, after incorporating the mediating variable, its direct association significantly increased (Effect = −0.063, 95% CI [−0.085, −0.042]). This indicates the presence of a typical suppression effect. A suppression effect is a significant statistical phenomenon in which the inclusion of a third variable in the model causes the direct effect of the independent variable on the dependent variable to either strengthen or reverse in direction. In such cases, the non-significant overall association often arises from multiple paths with opposing directions cancelling each other out at the aggregate level. A previous study illustrated this with the following example: suppose researchers examine the interrelationship between workers’ intelligence (X), levels of boredom (M), and the number of errors made during assembly line tasks (Y). It is reasonable to posit that, ceteris paribus, more intelligent workers make fewer errors, yet exhibit higher levels of boredom, which itself correlates positively with error counts. Thus, intelligence exerts a direct negative association on errors, while indirectly exerting a positive association via boredom as a mediator. Combined, these two hypothetical association may cancel each other out, resulting in a null overall association of intelligence on errors (MacKinnon et al., 2000). In this study, the association of rest intolerance on physical exercise is mediated simultaneously through two parallel yet oppositely directed pathways: on the one hand, rest intolerance diminishes exercise motivation, exerting a negative influence on physical activity through diminished motivation; on the other hand, it exerts a positive influence by enhancing exercise adherence levels. These pathways statistically form a “hedging” relationship: the negative pathway tends to reduce physical exercise, while the positive pathway promotes it. The opposing forces of these two pathways pull against each other, cancelling each other out and rendering the overall association non-significant. However, when decomposed, their respective significant association become clearly observable, ultimately masking the overall association. This finding reveals a dual mechanism through which rest intolerance influences health behaviours: it may foster superficial behavioural persistence as an external pressure, while simultaneously inhibiting sustained participation motivation by depleting psychological resources. Practically, this implies that merely reducing rest intolerance may not automatically promote physical exercise, as the phenomenon involves intertwined opposing psychological processes. Therefore, interventions should concurrently address reducing the attrition of psychological resilience and motivation caused by rest intolerance, while redirecting its potential “perseverance drive” towards healthier behavioural regulation. Future research may further track the dynamic equilibrium between these pathways through longitudinal designs or experimental methods, and explore how individual differences influence their relative strengths, thereby providing more robust theoretical support for tailored health behaviour interventions.

### 4.2 The mediating effect of exercise adherence and psychological resilience on rest intolerance and physical exercise levels

To examine the mediating effects of exercise adherence and psychological resilience on the relationship between rest intolerance and physical exercise, a bias-corrected bootstrap method was employed. Results indicated that exercise adherence significantly and positively mediated the relationship between rest intolerance and physical exercise (Effect = 0.065, 95% CI [0.047, 0.083]), supporting Hypothesis H2. This finding aligns with existing research: Dishman (1988) noted that individuals may maintain exercise behaviour to counter internalised anxiety or self-criticism, even under high psychological stress or negative emotional states. Similarly, Teixeira et al. (2012) proposed that exercise serves as an affective regulation strategy to alleviate guilt arising from perceptions of “not trying hard enough”. Rodrigues et al. (2018) demonstrated that individuals may exhibit high levels of exercise adherence even when motivation arises in non-fully autonomous contexts, particularly when exercise is perceived as a means of “compensation”. These studies collectively suggest that under rest intolerance motivation, individuals persist in exercise to mitigate negative self-evaluation—a behavioural response consistent with theoretical predictions and observable in practice. In comparison, although the mediating association of psychological resilience between rest intolerance and physical exercise was positive, it did not reach statistical significance (Effect = 0.002, 95% CI [−0.004, 0.007]), thus failing to support the hypothesis H3 that psychological resilience acts as an independent mediator. This outcome holds theoretical plausibility: psychological resilience constitutes a relatively stable psychological resource, typically developed through long-term life experiences and the accumulation of positive emotions. In contrast, rest intolerance represents situational social normative pressure, resulting in limited associative strength between the two constructs (Li et al., 2024; Huellemann et al., 2021). Although multiple studies indicate psychological resilience positively predicts physical exercise behaviour, its association sizes are typically modest and manifest more frequently in moderation or chained mediation pathways rather than as an independent direct mediator. For instance, a study involving 2,588 adolescents found that psychological resilience indirectly promoted sports participation by enhancing exercise motivation; however, its independent mediating effect was not prominent after controlling for other variables (Hu et al., 2025). In the present study, while psychological resilience also positively predicted physical exercise, its association size was comparatively limited relative to exercise adherence and exercise motivation, rendering its independent mediating pathway non-significant within the overall model. Exercise adherence demonstrated a more prominent mediating role, whereas psychological resilience played a supplementary function within the chained pathway. This suggests that the indirect prediction of physical exercise adherence by rest intolerance may be primarily manifested through behavioural-level “extrinsic persistence” rather than changes at the psychological resource level. This finding suggests that when addressing university students with high rest intolerance, enhancing psychological resilience alone may not directly promote their exercise behaviour. Instead, improving their exercise patterns should focus on modifying behavioural and motivational structures. Future research may further explore whether different types of shame (e.g., internalised shame, socially evaluated shame) exert differential association on psychological resilience, thereby providing a more nuanced psychological mechanism explanation for the model.

### 4.3 The chained mediating effects of exercise adherence and exercise motivation on rest intolerance and physical exercise levels

This study provides empirical validation of the significant chained mediating pathway: “rest intolerance → exercise adherence → exercise motivation → physical exercise” (Effect = −0.020, 95% CI [−0.026, −0.014]), supporting Hypothesis H4. This pathway suggests that while high rest intolerance may temporarily enhance exercise adherence, it subsequently diminishes exercise motivation, ultimately exerting a negative impact on physical activity. This finding aligns closely with the core tenets of Self-Determination Theory (Gu et al., 2021): when individuals are compelled to exercise due to feelings of shame, their autonomy and enjoyment diminish, shifting motivation from intrinsic to extrinsic—a pattern detrimental to long-term persistence (Johnston, 2022; Morrissy, 2011; Zhang et al., 2025). Contrary to Van Roie et al. (2015) finding that ‘extrinsic motivation can also promote exercise,’ this study demonstrates that under rest intolerance conditions, extrinsic regulation struggles to sustain enduring health behaviours. This discrepancy may stem from the interactive influence of measurement tools and cultural contexts: the MPAM-R scale employed in this study places greater emphasis on the internalisation of motivation, whereas “compensatory exercise” within East Asian cultures often carries connotations of self-punishment, readily inducing motivational fatigue and behavioural burnout (Lin et al., 2022). From a developmental perspective, university students are at a critical stage of self-identity formation. Exercise behaviours regulated by external factors may conflict with their internal value systems, leading to failed exercise motivation. The discovery of this chained mediating effect provides crucial insights into the mechanisms through which rest intolerance influences physical exercise. Future research may employ longitudinal designs to track shifts in motivational trajectories while examining the mediating role of fundamental psychological needs. Incorporating neurophysiological indicators such as heart rate variability and cortisol levels could further elucidate the physiological mechanisms through which rest intolerance influences motivational processes.

### 4.4 The chained mediating effect of psychological resilience and exercise motivation on rest intolerance and physical exercise levels

This study validated the chained mediating pathway of ‘rest intolerance → psychological resilience → exercise motivation → physical exercise’. Although the association was significant, the overall association size was small (Effect = 0.003, 95% CI [0.002, 0.003]), supporting Hypothesis H5. This finding suggests that although rest intolerance has a weaker influence on psychological resilience, the chain of events whereby psychological resilience enhances exercise motivation and subsequently promotes physical activity remains valid. That is, university students with higher psychological resilience are more likely to experience more positive and autonomous exercise motivation, and this motivation facilitates an increase in their physical exercise levels. This aligns closely with the core tenets of self-determination theory. Self-Determination Theory emphasises that the sustainability of human behaviour depends on the degree of motivation’s “autonomy”—that is, the transition from external to internal regulation (Standage and Ryan, 2020). This also resonates with prior research grounded in resource conservation theory and the dual-factor model of mental health. Such studies revealed that physical exercise exerts dual association on university students’ mental health through a chain of mediators involving mindfulness and psychological resilience. Here, psychological resilience functions as a pivotal psychological resource, aligning with perspectives that suggest psychological resilience primarily exerts protective association under stress (Guo, 2025). However, the relatively weak influence of rest intolerance on psychological resilience resulted in a limited association size for the entire chain of pathways. This characteristic aligns with the theoretical positioning of psychological resilience as a stable psychological trait: trait resilience relies more on an individual’s long-term accumulation of resources than on the short-term impact of a single emotion or normative pressure (Li et al., 2024). Moreover, the significance of the chained mediating pathway indirectly underscores the central role of motivation within the model: regardless of an individual’s psychological resource levels, physical exercise behaviour is ultimately shaped by the quality of motivation and internalisation. This aligns strongly with Self-Determination Theory’s emphasis on ‘the importance of transitioning from external to internal regulation’ (Standage and Ryan, 2020). A series of prior studies have consistently found that adolescents’ participation in physical activity is jointly influenced by ‘perceived self-worth in sport’ and ‘psychological resilience,’ with their mechanisms of action mediated through the variables of ‘exercise motivation’ and ‘social support.’ These studies collectively point to a conclusion: any intervention aimed at promoting physical exercise must prioritise the stimulation and maintenance of high-quality intrinsic motivation as its primary objective (Tang et al., 2025; Peng et al., 2025). Future research may adopt a multi-pathway approach to further investigate whether different dimensions of psychological resilience (such as emotional regulation, positive cognition, and interpersonal support) exert differential association on distinct motivational types. Concurrently, experimental or longitudinal data should be employed to validate how psychological resilience promotes the internalisation of motivation over the long term, thereby providing a more comprehensive understanding of its operational mechanisms within exercise behaviour.

### 4.5 Limitations and recommendations

This study employed an online questionnaire to collect data. Although the sample size was substantial and coverage extensive, online recruitment may introduce self-selection bias. Furthermore, as specific response rates from individual institutions were unavailable, it is impossible to ascertain whether participation was balanced across different universities or regions. Consequently, the sample may exhibit uneven distribution at the regional or institutional level. Although the demographic characteristics of this study’s sample closely approximate those of the broader undergraduate population in Chinese higher education institutions, it cannot be regarded as strictly nationally representative. Consequently, conclusions drawn from this data should be interpreted cautiously, taking into account the limitations inherent in the sampling methodology. Furthermore, the cross-sectional design employed in this study introduces temporal ambiguity regarding the causal sequence and directionality between variables. It is therefore impossible to definitively establish whether rest intolerance precedes changes in physical exercise levels. For instance, reverse or bidirectional causality may exist: lower physical activity levels could lead to increased negative self-evaluation, guilt, and self-criticism, thereby reinforcing rest intolerance. Conversely, reduced exercise may diminish positive emotions and self-efficacy, making individuals more susceptible to feelings of ‘undeserving rest’. Similarly, psychological resilience may result from physical exercise while simultaneously influencing exercise behaviour, rather than being solely predicted unidirectionally by rest intolerance. Given the cross-sectional nature of the data, this study cannot rule out these potential reverse or bidirectional pathways. Consequently, the model presented here is better suited as a foundation for testing theoretical mechanisms rather than as a strictly causal model.

All variables in this study were measured using self-report methods, which may be subject to common method bias or social desirability association. Although Harman’s single-factor test indicated that common method bias was not severe, future research is advised to incorporate objective measures, such as accelerometer-monitored physical activity, sleep trackers, or daily behavioural logs, to further enhance ecological validity. Moreover, the present model did not include potential confounding factors such as academic stress, social support, or cultural values, which may interact with rest intolerance and physical activity behaviour. Future studies could construct more comprehensive structural models to validate these relationships.

Finally, future research should adopt more causally inferential designs to test the proposed mechanisms. Firstly, longitudinal designs with multiple time points could employ cross-lagged models to clarify temporal relationships among rest intolerance, exercise motivation, psychological resilience, and physical exercise, thereby testing bidirectional or dynamic mechanisms. Secondly, causal inference could be strengthened through experimental or intervention studies. For instance, experimental conditions such as rest norm activation, rest intolerance mitigation training, or motivation internalisation enhancement courses could be implemented to observe the immediate and delayed association on physical activity following the manipulation of rest intolerance or motivation. Moreover, randomised controlled trials implementing structured physical participation programmes, psychological resilience enhancement training, or self-compassion courses could systematically validate the psychological mechanisms underlying rest intolerance pathways. Through these approaches, future research will more accurately elucidate the dynamic influence of rest intolerance on university students’ exercise behaviour, providing robust empirical foundations for developing targeted behavioural promotion strategies.

## 5 Conclusion

This study employed a large-scale national sample to construct and test a dual-path chain mediation model examining how rest intolerance influences university students’ physical exercise. Findings revealed that rest intolerance positively correlates with exercise adherence and exercise motivation, while negatively correlating with psychological resilience and physical exercise. Exercise adherence, psychological resilience, and exercise motivation mediated the relationship between university students’ rest intolerance and physical exercise, with the two chain mediation pathways exhibiting opposite directions. It is recommended that exercise promotion interventions for students with high rest intolerance should concurrently address the sources of behavioural persistence and the preservation of motivational and psychological resources. This approach prevents motivational depletion arising from compensatory persistence and fosters more sustainable participation in physical activity.

## Data Availability

The raw data supporting the conclusions of this article will be made available by the authors without undue reservation.
