# Supplementary material for: How does rest intolerance affect physical exercise? A dual-pathway mediation model of exercise adherence and psychological resilience
Source: Front Psychol. 2026 Jan 6;16:1723996. doi: 10.3389/fpsyg.2025.1723996 (PMC12816276; doi:10.3389/fpsyg.2025.1723996)
Supplement: Supplementary file 1 [file Supplementary_file_1.docx]

**Appendix: Detailed Item Information for the Scale**

**(1) Rest Intolerance**

| No. | Question | 1 | 2 | 3 | 4 | 5 |
| --- | --- | --- | --- | --- | --- | --- |
|  |  | Not at all | Not quite | Not clear | Comparatively | Exactly |
| 1 | I feel guilty when I take time off or have fun | □ | □ | □ | □ | □ |
| 2 | I feel like a failure when I rest or play | □ | □ | □ | □ | □ |
| 3 | I worry about being surpassed by others when I rest or play | □ | □ | □ | □ | □ |
| 4 | When I rest, I always think of peers who work harder than me | □ | □ | □ | □ | □ |
| 5 | I can’t help but think of studying or working when I rest or play | □ | □ | □ | □ | □ |
| 6 | I always feel like there are other things I haven’t done when I rest or play | □ | □ | □ | □ | □ |
| 7 | I think I should spend more time studying and working rather than resting | □ | □ | □ | □ | □ |
| 8 | I think I should use my time off for more valuable things | □ | □ | □ | □ | □ |

**Table 1** **Rest Intolerance Scale-8**

**(2) Physical Exercise**

**Table 2 Physical Exercise Level Scale**

| What is the intensity of your physical exercise? | □Light exercise (such as walking, doing radio exercises, playing gate ball, etc.)  □Low intensity and less intense sports (recreational activities such as playing volleyball, table tennis, jogging, etc.)  □Moderate intensity, intense and prolonged exercise (such as cycling, running, playing table tennis)  □High intensity but not lasting sports (such as play badminton, volleyball, etc.) with shortness of breath and a lot of sweat  □Intense and sustained exercise with rapid breathing and heavy sweating (such as running, aerobics routines, swimming, etc.) |
| --- | --- |
| How many minutes do you engage in the aforementioned intensity sports activities? | □Less than 10 minutes  □11 to 20 minutes  □21 minutes to 30 minutes  □31 minutes to 59 minutes  □more than 60 minutes |
| How many times have you engaged in the aforementioned sports activities? | □Less than once a month  □2-3 times a month  □1-2 times a week  □3-5 times a week  □Approximately 1 activity per day |

**(3) Exercise Adherence Scale**

| **No.** | **Question** | 1 | 2 | 3 | 4 | 5 |
| --- | --- | --- | --- | --- | --- | --- |
|  |  | Not at all | Not quite | Not clear | Comparatively | Exactly |
| 1 | I participate in physical activity for at least 1 hour at a time | □ | □ | □ | □ | □ |
| 2 | It has been 6 months since I started physical activity | □ | □ | □ | □ | □ |
| 3 | I am physically active at least 3 times a week | □ | □ | □ | □ | □ |
| 4 | I am used to working out when it's time to work out | □ | □ | □ | □ | □ |
| 5 | I will try to achieve a certain amount of exercise each time | □ | □ | □ | □ | □ |
| 6 | I will put more effort into practising new sports skills to improve myself. | □ | □ | □ | □ | □ |
| 7 | I will do my best every time, whether I like it or not. | □ | □ | □ | □ | □ |
| 8 | I am committed to investing time, money and energy in physical activity. | □ | □ | □ | □ | □ |
| 9 | I am willing to set aside a relatively regular amount of time for physical activity. | □ | □ | □ | □ | □ |
| 10 | I will try my best to avoid interruptions to do physical exercise | □ | □ | □ | □ | □ |
| 11 | I like the feeling of physical activity | □ | □ | □ | □ | □ |
| 12 | I enjoy working out with other people. | □ | □ | □ | □ | □ |
| 13 | I often feel positive after working out | □ | □ | □ | □ | □ |
| 14 | I often feel strong after a workout | □ | □ | □ | □ | □ |
| 15 | I feel good physically and mentally after a workout | □ | □ | □ | □ | □ |

**Table 3 Exercise Adherence Scale**

**(4) Psychological resilience**

**Table 4 Adolescent Mental Toughness Scale**

| **No.** | **Question** | 1 | 2 | 3 | 4 | 5 |
| --- | --- | --- | --- | --- | --- | --- |
|  |  | Not at all | Not quite | Not clear | Comparatively | Exactly |
| 1 | Failure always discourages me. | □ | □ | □ | □ | □ |
| 2 | I have a hard time controlling my unpleasant emotions. | □ | □ | □ | □ | □ |
| 3 | I have clear goals in my life. | □ | □ | □ | □ | □ |
| 4 | I am generally more mature and experienced after experiencing setbacks. | □ | □ | □ | □ | □ |
| 5 | Failures and setbacks can make me doubt my abilities. | □ | □ | □ | □ | □ |
| 6 | I can't always find someone to talk to when something unpleasant happens to me. | □ | □ | □ | □ | □ |
| 7 | I have a friend my own age to whom I can tell my difficulties. | □ | □ | □ | □ | □ |
| 8 | My parents respect my opinion. | □ | □ | □ | □ | □ |
| 9 | I don't know who to go to when I am in trouble and need help. | □ | □ | □ | □ | □ |
| 10 | I think the process of things helps one grow more than the outcome. | □ | □ | □ | □ | □ |
| 11 | When I face difficulties, I usually make a plan and a solution. | □ | □ | □ | □ | □ |
| 12 | I am used to keeping things inside rather than talking to people. | □ | □ | □ | □ | □ |
| 13 | I think adversity has a motivating effect on people. | □ | □ | □ | □ | □ |
| 14 | Adversity is sometimes an aid to growth. | □ | □ | □ | □ | □ |
| 15 | My parents always liked to interfere with my ideas. | □ | □ | □ | □ | □ |
| 16 | At home, no one always listens to what I say. | □ | □ | □ | □ | □ |
| 17 | My parents lack confidence and moral support for me. | □ | □ | □ | □ | □ |
| 18 | I take it upon myself to talk to someone when I am in trouble. | □ | □ | □ | □ | □ |
| 19 | My parents are never harsh on me. | □ | □ | □ | □ | □ |
| 20 | I concentrate all my energy when facing difficulties. | □ | □ | □ | □ | □ |
| 21 | It usually takes me a long time to forget unpleasant things. | □ | □ | □ | □ | □ |
| 22 | My parents always encourage me to do my best. | □ | □ | □ | □ | □ |
| 23 | I can adjust my emotions very well in a short time. | □ | □ | □ | □ | □ |
| 24 | I will set goals for myself to push myself forward. | □ | □ | □ | □ | □ |
| 25 | I think everything has its positive side. | □ | □ | □ | □ | □ |
| 26 | I am not willing to tell others when I am in a bad mood. | □ | □ | □ | □ | □ |
| 27 | I have big mood swings and am prone to ups and downs | □ | □ | □ | □ | □ |

**(5) Exercise Motivation**

**Table 5 Motives for Physical Activity Measure–Revised**

| I exercise because… | 1 | 2 | 3 | 4 | 5 |
| --- | --- | --- | --- | --- | --- |
|  | Not at all | Not quite | Not clear | Comparatively | Exactly |
| (Health) I wish to maintain physical fitness | □ | □ | □ | □ | □ |
| (Appearance) I wish to maintain or improve my physique | □ | □ | □ | □ | □ |
| (Enjoyment) I wish to maintain a cheerful disposition | □ | □ | □ | □ | □ |
| (Ability) I wish to enhance my current athletic performance | □ | □ | □ | □ | □ |
| (Social) I wish to strengthen friendships and bonds | □ | □ | □ | □ | □ |

**Descriptive Statistics for Physical Exercise**

**Table 1 Descriptive Data on Physical Exercise**

| **Median** | | 12.000 |
| --- | --- | --- |
| **Skewness** | | 1.918 |
| **Standard error of skewness** | | 0.015 |
| **Kurtosis** | | 3.663 |
| **Standard error of kurtosis** | | 0.031 |
| **Percentile** | **25** | 5.000 |
|  | **50** | 12.000 |
|  | **75** | 24.000 |


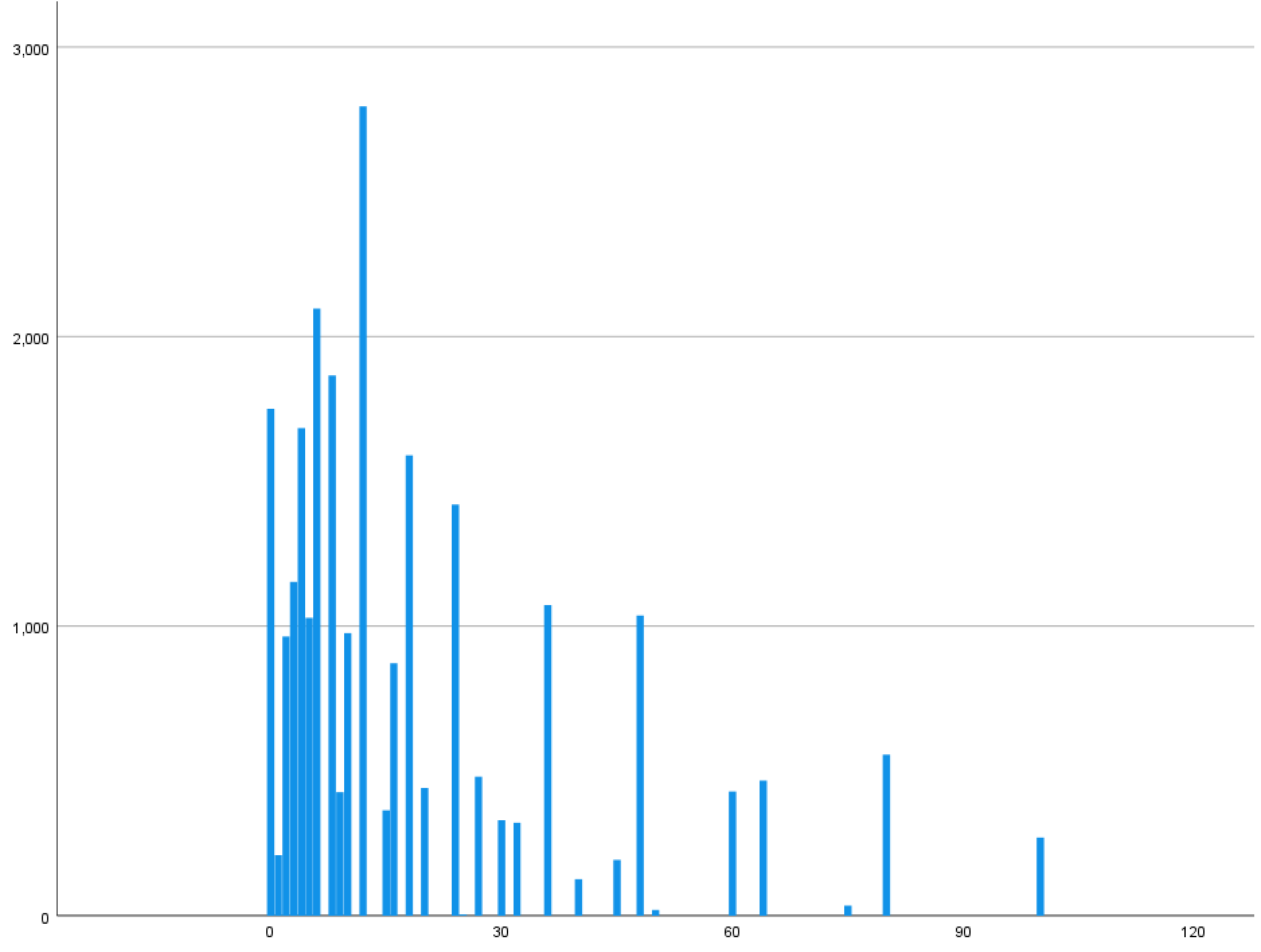


Note: The X-axis represents the score for physical exercise, while the Y-axis denotes frequency.

**Figure 1 Histogram of Physical Exercise Score Distribution**
